# One-Dimensional Shaving-like BiVO_4_ Nanobelts: Synthesis, Characterization and Photocatalytic Activity with Methylene Blue

**DOI:** 10.3390/molecules28237793

**Published:** 2023-11-27

**Authors:** Yuling Liu, Shengxia Duan, Jian Liu, Xiaomin Jin, Fengqiang Dong, Guangge Shi, Qingsheng Wu

**Affiliations:** 1 College of Chemistry and Chemical Engineering, Heze University, Daxue Road No. 2269, Heze 274015, China; liuyuling@hezeu.edu.cn (Y.L.); duanshengxia@hezeu.edu.cn (S.D.); 17661369763@163.com (X.J.); 2 College of Agriculture and Bioengineering, Heze University, Daxue Road No. 2269, Heze 274015, China; 3 Department of Chemistry, TongJi University, Siping Road No. 1239, Shanghai 200092, China; dongfengqiang@bjguozhi.cn; 4 Food and Drug Inspection and Testing Institute of Heze, Taihu Road No. 1999, Heze 274015, China; ss6265@163.com

**Keywords:** oxide hydrothermal method, shaving-like BiVO_4_ nanobelts, optical properties, electronic properties, MB degradation

## Abstract

One-dimensional shaving-like BiVO_4_ nanobelts were successfully synthesized via the oxide hydrothermal method (OHS), using V_2_O_5_ and Bi_2_O_3_ as raw materials and PEG 10000 (polyethylene glycol 10000) as a template. Multiple techniques, including XRD, SEM, TEM, HRTEM, UV–Vis, XPS, and photoelectrochemical measurements, were applied to characterize the obtained materials. The thickness of the BiVO_4_ nanobelt was approximately 10 nm, while the width was approximately 500 nm. EIS results showed that visible-light illumination caused the photogenerated charge of the BiVO_4_ nanobelts to have a faster transfer and a higher separation efficiency. Photocatalytic experiments indicated that with BiVO_4_ nanobelts as a catalyst, the degradation rate of MB (methylene blue) was close to 92.4%, and it disintegrated after two hours. Moreover, the pseudo-first-order kinetic model can be used to describe the photodecomposition reaction of MB catalysed by BiVO_4_ nanobelts. And this excellent photocatalytic activity of the shaving-like BiVO_4_ nanobelts may be related to their special morphology, narrow band gap (~2.19 eV), faster transfer and the separation efficiency of the photogenerated charge, leading to strong absorption in the visible region and improving the separation of the photogenerated electron–hole pairs. These novel monoclinic BiVO_4_ nanobelts exhibited great photocatalytic activity and are thus a promising candidate for application in visible-light-responsive photocatalysts.

## 1. Introduction

Of the various kinds of pollutants causing water pollution, the widespread presence of organic dyes in industrial wastewater is becoming increasingly hazardous in our environment and has become the focal point of future research studies on waste water treatment, since dyes are increasingly used in the textile, leather, paper, rubber, plastics, cosmetics, pharmaceutical, and food industries [1]. Hence, it is important to remove or destroy these organic dyes before they are discharged into the environment. However, traditional processing methods, including adsorption, the membrane filter technique and coagulation, are ineffective for decoloring such wastewaters. As a result, new technologies with more efficiency and which use less energy have stimulated intensive research. An alternative to traditional processing methods is the photocatalytic method, since it produces no secondary pollution and is highly convenient, and low cost. In addition, this photocatalysis method is based on the generation of very reactive species such as hydroxyl radicals (·OH), which could nonselectively oxidize a broad range of organic pollutants with high efficiency [2].

In recent decades, several bismuth-based complex oxides such as Bi_2_O_3_, Bi_2_Sn_2_O_7_, and Bi_2_MO_6_, have been regarded as significant in the field of photocatalysisas. Among them, BiVO_4_, an *n*-type semiconductor photocatalyst with a direct band gap of 2.4 eV has great absorption properties in the presence of visible-light irradiation. Recent studies showed that BiVO_4_ has excellent photocatalytic and photophysical properties under visible light [3,4,5], and it has attracted increasing amounts of attention for the degradation of organic pollutants, oxygen evolution from water splitting and CO_2_ conversion because of its small band gap energy, nontoxicity, high stability and visible-light absorbance [6,7,8,9,10]. On the other hand, the structure of BiVO_4_ has a significant impact on its photocatalytic performance. Generally speaking, BiVO_4_ is a polycrystalline compound, which has three main crystal types: tetragonal scheelite (s-t BiVO_4_), a tetragonal zirconium silicate type (z-t BiVO_4_) and a monoclinic deformed scheelite type (s-m BiVO_4_) [11,12].

Therefore, great efforts have been made to prepare BiVO_4_ with various crystal types to investigate their photocatalytic performance. For instance, Lin et al. prepared eight crystalline phases of BiVO_4_ using a simple solution-based hydrothermal method and demonstrated great photocatalytic performance for CV (crystal violet) degradation [13]. Trinh, D.N. et al. prepared monoclinic–tetragonal heterostructured BiVO_4_ via the facile solvothermal method and applied it for RhB (Rhodamine B) photodegradation [14]. Nguyen et al. prepared BiVO_4_ using a facile solvothermal router and this BiVO_4_ material showed high activity in the photodecomposition of RhB [15]. Li and his co-workers successfully prepared mulberry-like BiVO_4_ architectures via a facile solvothermal route [16]. Although abundant BiVO_4_ materials with various crystal types and synthetic methods have been obtained, they still have flaws, such as uneconomic synthesis methods, heterogeneous morphology and poor catalytic efficiency. Hence, it is still necessary to explore new BiVO_4_ materials with gentler synthesis methods and better photocatalytic properties.

In this study, BiVO_4_ nanobelts of the monoclinic deformed scheelite type were successfully prepared by using V_2_O_5_ and Bi_2_O_3_ as raw materials and the one-step hydrothermal method under the control of a PEG 10000 surfactant. And the synthesized BiVO_4_ nanobelts were characterized well via XRD, FT-IR, FE-SEM, TEM, HR-TEM and XPS. Herin, methylene blue (MB) was selected as a model organic contaminant since it not only has a stable molecular structure but is also applied extensively in all kinds of textile production. Moreover, it is a heterocyclic aromatic chemical compound with the IUPAC name 3,7-bis(dimethylamino)-phenothiazin-5-ium chloride and molecular formula C_16_H_18_N_3_SCl, and it is a highly toxic chemical primarily used as a dye, which can bring about negative effects, such as vomiting, tissue necrosis, increased heart rate and shock in humans. Furthermore, photoelectrochemical measurements of BiVO_4_ nanobelts were carried out using an electrochemical workstation and their photocatalytic properties were investigated via the degradation of an MB solution under simulated visible-light irradiation.

## 2. Results and Discussion

### 2.1. Structure and Morphology

#### 2.1.1. The FTIR Spectrum (a) and XRD Pattern (b) of the BiVO_4_ Sample

**Figure 1:**
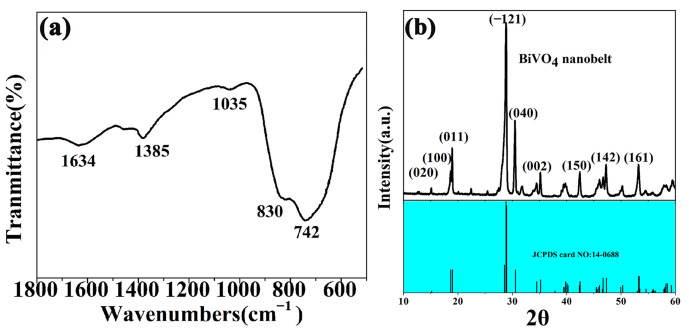
The FTIR spectrum (**a**) and XRD pattern (**b**) of the BiVO_4_ sample.

Figure 1a shows the infrared spectrum of the BiVO_4_ sample at 200 °C for 24 h. The peaks with wave counts of 742 cm^−1^ and 1035 cm^−1^ can be attributed to the asymmetric contraction of VO_4_^3−^, while the absorption peak at 830 cm^−1^ can be ascribed to ν1 symmetric contraction peak of VO_4_^3−^. Moreover, the small absorption peaks at 1634 cm^−1^ and 1385 cm^−1^ are the characteristic absorption peaks of H-O-H in water. These characteristic absorption peaks are consistent with those reported in previous studies, indicating the feasibility of using oxides as a reaction material to synthesize vanadate [17,18].

The XRD pattern is an important basis to confirm the purity and crystal structure of BiVO_4_. As can be seen from Figure 1b, the XRD pattern of BiVO_4_ is consistent with the standard JCPDS card (No. 14-0688), indicating the high purity of the obtained material. Moreover, the cell parameters calculated using Jade were a = 5.205, b = 11.61, and c = 5.097; β = 90.20, which was close to the standard values a = 5.195, b = 11.70, c = 5.092; β = 90.38, further confirming the successful preparation of BiVO_4_. Additionally, though BiVO_4_ (s-m) and BiVO_4_ (z-t) both have a scheelite structure, the important distinguishing feature between BiVO_4_ (s-m) and BiVO_4_ (z-t) is whether there are splitting peaks at 2θ = 18.5°, 35° and 46°. As can be seen from Figure 1b, there are distinct splitting peaks in the characteristic regions 2θ = 18.5 and 46°, indicating the s-m crystal type of BiVO_4_. Furthermore, the primary particle size calculated from the strongest diffraction peak (−1 2 1) is 19.5 nm. The XRD patterns clearly showed that the monoclinic BiVO_4_ prepared by OHS was of great significance because of its lower reaction temperature [19,20].

#### 2.1.2. FE-SEM and TEM

**Figure 2:**
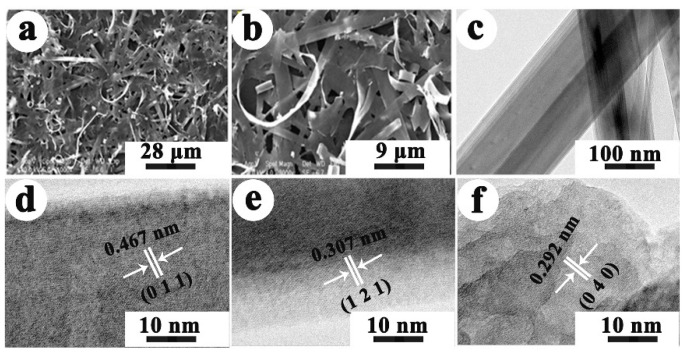
SEM (**a**,**b**), TEM (**c**) and HRTEM images (**d**–**f**) of the BiVO_4_ sample.

As can be seen from Figure 2a–c, the obtained BiVO_4_ sample displayed the morphology of a nanobelt with a uniform size, high aspect ratio, and ultra-thin structure. The thickness of the nanobelt is approximately 10 nm while the width is approximately 80 nm. Since the nanobelts are so long and entangled, the length of the nanobelts cannot be displayed accurately in SEM images. The formation of nanobelts can be ascribed to the application of linear molecular PEG, utilized as a morphology regulator, which was beneficial to the growth of BiVO_4_ along the one-dimensional direction in the reaction system. The possible conversion mechanism of nanobelts was given in the following discussion. Figure 2d–f shows the HR-TEM image of the BiVO_4_ sample. The lattice fringes with spacings of 0.467 nm, 0.307 nm, and 0.292 nm were ascribed to the (0 1 1), (1 2 1) and (0 4 0) planes of BiVO_4_, respectively. This result clearly indicated that BiVO_4_ nanobelts had been successfully synthesized in company with XRD analysis.

#### 2.1.3. N_2_ Adsorption–Desorption Isotherms and Diffuse Reflectance Spectroscopy

**Figure 3:**
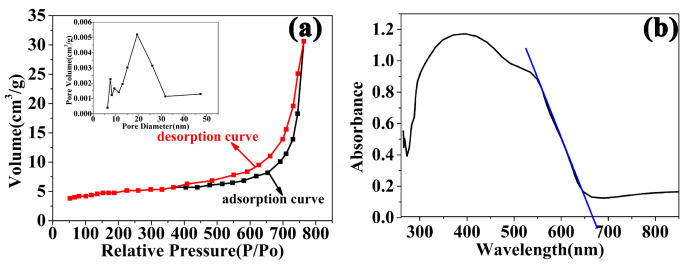
N_2_ adsorption–desorption isotherm with the corresponding pore size distribution (**a**) and diffuse reflectance spectroscopy (**b**) of the BiVO_4_ sample prepared by OHS at 200 °C.

N_2_ adsorption–desorption isotherm measurements were carried out to evaluate the specific surface area and the average pore size distributions of the obtained BiVO_4_ sample. According to the Brunauer–Deming–Deming–Teller (BDDT) classification, the isotherms can be nearly categorized as type IV. Figure 3a dispalyed the N_2_ adsorption–desorption isotherm and corresponding pore distributions for BiVO_4_ nanobelts. The BET-specific surface area of this nanobelt is 9.05 m^2^/g while its average pore size was 19.2 nm calculated by the BJH (Barett–Joyner–Halenda) formula [21], displaying a large number of pores not more than 20 nm in these nanobelt, suggesting its mesoporous structures.

In order to determine the optical absorption properties of BiVO_4_ nanobelts, UV–Vis diffuse reflectance measurements were performed at a certain wavelength range between 200 and 800 nm. Figure 3b displayed the UV–Vis diffuse reflectance spectra of BiVO_4_ nanobelts. It can be seen that shaving-like BiVO_4_ nanobelts not only have strong ultraviolet absorption properties, but also show good absorption properties in the visible region, which is an important characteristic of monoclinic BiVO_4_ materials. Moreover, the maximum absorption wavelength in the visible region is approximately 565 nm, shown in Figure 3b. The energy gap of BiVO_4_ nanobelts was approximately 2.19 eV by using E = 1240/λ eV [22]. Furthermore, based on a previous study [3], the valence band of monoclinic BiVO_4_ not only has an O 2p orbit, but also Bi 6s, thus, the band gap of BiVO_4_ prepared in this study is much smaller than that of tetragonal BiVO_4_ (2.9 eV). In addition, the monoclinic BiVO_4_ may have high visible-light photocatalysis capability because of the good mobility of the photocarrier and its lower band gap by the mixed valence band [23].

#### 2.1.4. XPS Measurement

**Figure 4:**
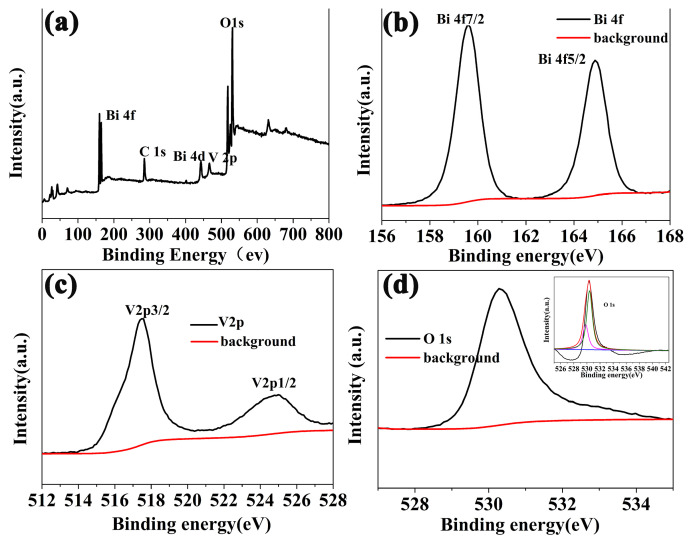
XPS spectra of the BiVO_4_ nanobelts: (**a**) XPS survey spectrum, (**b**) high-resolution Bi 4f spectrum, (**c**) high-resolution V 2p spectrum, and (**d**) high-resolution O 1s spectrum with fitted curves inset.

XPS measurement was performed to explore the chemical compositions and valence states of the BiVO_4_ nanobelts, and the XPS spectra are shown in Figure 4. Figure 4a shows the survey XPS spectrum of the BiVO_4_ nanobelts in the range of 0–800 eV, which exhibited the predominant elements of Bi, V, O and C. The presence of a C 1s peak (284.8 eV) can be attributed to the adventitious carbon on the sample’s surface, which originated from the decomposition of PEG 10000 in the hydrothermal reaction at 200 °C. The carbon was not completely washed away during the sample washing process, leading to the presence of C 1s peak in the XPS spectrum. Figure 4b shows the Bi 4f high-resolution XPS spectrum. The two peaks were centered at 159.6 eV and 164.9 eV with an interval of 5.3 eV associated with the Bi 4f 5/2 and Bi 4f 7/2, separately. Figure 4c shows the high-resolution XPS spectrum of V 2p. Peaks with 7.3 eV apart occurring at 524.8 eV and 517.5 eV are associated well with V 2p 1/2 and V 2p 3/2, respectively. Figure 4d shows the O 1s high-resolution spectrogram with its corresponding fitting curves. Two peaks centered at 530.3 eV and 531.8 eV can be well ascribed to oxygen in the lattice of BiVO_4_ nanobelts and adsorbed oxygen by BiVO_4_ materials, respectively. All the above structural results prove that the BiVO_4_ nanobelts have been synthesized successfully [24].

### 2.2. Possible Synthesis Mechanism

The BiVO_4_ nanobelts were synthesized according to the equation below and through the five-step process: ball milling, hydration, dehydration, nucleation, and growing.

$${\mathrm{Bi}}_{2}O_{3}+V_{2}O_{5}\overset{H_{2}O,\;\mathrm{PEG}\;10000}{\underset{200\;{}^{\circ}C}{\to }}2{\mathrm{BiVO}}_{4}$$

**Scheme 1:**
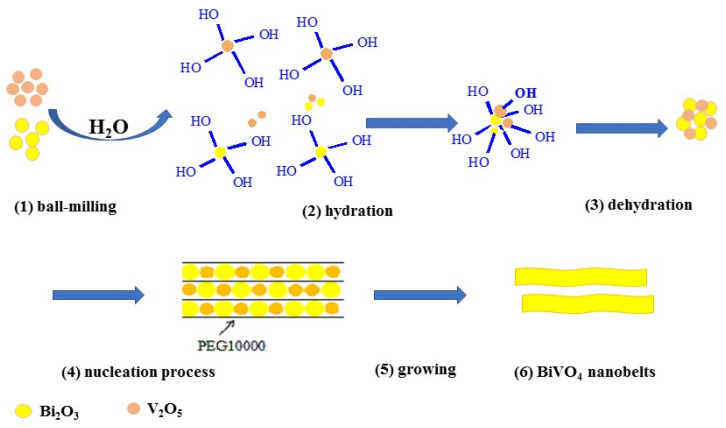
Schematic diagram of the BiVO_4_ nanobelt synthesis process.

As shown in Scheme 1, the formation of products followed the possible basic process of ball milling, hydration, dehydration, nucleation, and growing. Firstly, the collision and friction between the V_2_O_5_ and Bi_2_O_3_ particles gradually became smaller under the atmosphere of faster-moving water vapor in an airtight hydrothermal reaction system, which were then uniformly dispersed in the reaction system. In the above procedure, every small solid particle can be considered as a small ball, which then formed countless ball milling rotors. Since this ball milling rotor was the reactant itself, this dispersal course can be named ball grinding. Secondly, the surface energy and the activity of each particle increased rapidly with the decrease in particle size. Moreover, numerous hydrated ions (the hydration process) were formed between the small particles and H_2_O molecules in order to reduce its surface activity. During the movement of hydrated ions, a series of processes occurred, including further friction, collision, dehydration, rehydration, separation and cross-linking. Thirdly, dehydration occurred (the dehydration process) when two hydrated ions collided or interacted with each other, eventually forming BiVO_4_ molecules. The number of BiVO_4_ molecules increased gradually and formed nucleation after reaching supersaturation (nucleation process), which then developed into microcrystals through the dissolution–deposition mechanism and grew into the target crystals, eventually. In the above procedures, PEG 10000 acted as a soft template during the growth of nuclei and forced nuclei to cluster in a one-dimensional direction, forming shaving-like BiVO_4_ nanobelts (growing) [25,26].

### 2.3. Photoelectrochemical Analyses

**Figure 5:**
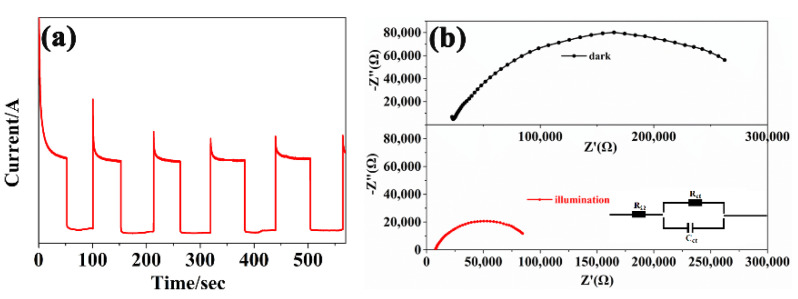
The transient photocurrent responses (**a**) and EIS Nyquist plots (dot) (**b**) of BiVO_4_ nanobelts.

Figure 5 shows the instantaneous photoelectric current and EIS Nyquist plots (dot) of BiVO_4_ nanobelts. Figure 5a displayed the several on–off cycles of intermittent illumination. As shown in Figure 5a, the photocurrent of BiVO_4_ nanobelts reached a constant value once under illumination; in contrast, the photoelectric current dropped to zero when illumination was removed, which revealed the good reproducibility of each cycle. There was an anodic spike of the photoelectric current curve when the light started illumination. After the current reached the peak value, it began to decline continuously until a constant photocurrent was achieved. This decline in photocurrent could be ascribed to the recombination of photoinduced carriers. During the decline in photocurrent, the holes were competitively recombined with electrons instead of being captured by reduced agents in the electrolyte. After the equilibration of competitive separation and recombination of electron–hole pairs, the photoelectric current became constant. The instantaneous photoelectric current can be used to describe the separation properties of photogenerated electrons and holes in the process of photocatalytic degradation indirectly [27].

Moreover, the EIS (electrochemical impedance) is also a proven analytical method for improving the efficiency of electron transport. As shown in Figure 5b, the impedance radius of the BiVO_4_ sample was significantly smaller under illumination than that in the dark. This proved that fewer electrons can pass through the interface of electrolyte without light [28]. In addition, the EIS Nyquist plots of BiVO_4_ nanobelts showed that there was only one semicircle both in the dark and in the light, which can be associated with the Randles equivalent circuit model (inset of Figure 5b) [29]. The electrical resistance R_Ω_ was related to charge transfer capacity, including the electrical resistance of wire connections, semiconductor catalysts and the electrolyte in the whole line. The C_ct_ and R_ct_ were related to the transfer of charge at the junction between photoelectrode and electrolyte. The smaller impedance radius of the BiVO_4_ sample under visible-light illumination indicated that photogenerated charges were transmitted faster and resulted in rapid separation of hole and electron, which was consistent with the high photocatalytic performance.

### 2.4. The Performance of the Photocatalysis

As a photocatalyst, BiVO_4_ has a strong ability of oxidation, which can lead to the degradation of most organic compounds. The photocatalytic degradation ability of BiVO_4_ nanobelts was evaluated by using a representative dye MB as the target molecule. The photocatalytic tests were carried out in a reaction chamber equipped with a cooling water cycle system to obtain a constant temperature. The light source was from a tungsten lamp (450 W) equipped with a UV cut-off filter to obtained light with a wavelength above 420 nm. The lamp was put in a cold trap to reduce the released energy. In the the photocatalysis experiment, 30 mg of the obtained BiVO_4_ material was scattered in 45 mL MB (50 mg/L) aqueous solution with magnetic stirring for 2 h in the dark to achieve an adsorption–desorption equilibrium.

**Figure 6:**
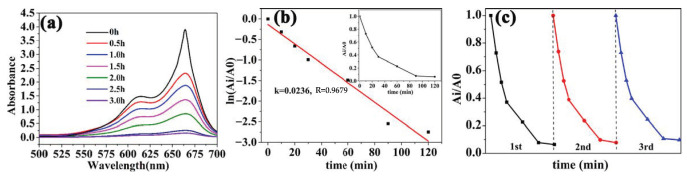
Corresponding time-dependent absorption spectra of the solution of MB in the presence of BiVO_4_ samples (**a**), the kinetics of photocatalytic degradation of MB over BiVO_4_ nanobelts with the degradation ratio inset (**b**), and cycling runs for the photodegradation of MB over BiVO_4_ samples (**c**).

As illustrated in Figure 6a, with the progress of photocatalytic degradation, the corresponding absorption peak intensity at 665 nm and 296 nm of MB gradually decreased and shifted to a shorter wavelength with prolongation of the irradiation time, which was due to the removal of the N-methyl group from MB molecules, revealing that the chromophoric structure of the dye was stepwise destroyed. In addition, the degradation ratio of MB solution is nearly 92.4% after photodegradation for 2 h, indicating the excellent photocatalytic performance of the obtained BiVO_4_ nanobelts.

The degradation ratio of the MB was calculated by the ratio of the instantaneous concentration to the initial concentration. In the following equations: η = (1 − C_t_/C_0_) × 100% and C_i_/C_0_ = A_i_/A_0_, C_0_ and A_0_ stand for the initial concentration of the MB in the solution and the initial absorbance of the solution, respectively. C_t_ and A_t_ symbolize the concentration of MB in the solution after the reaction for t min and the absorbance of the solution after irradiation for t min, respectively. The kinetics of the photocatalytic degradation of MB over BiVO_4_ nanobelts can be gained through the linear correlation between ln(A_t_/A_0_) and the degradation time (t, min). According to Figure 6b, it can be seen that the linear correlation between ln(A_t_/A_0_) and time is good. The kinetics constant and the Adj. R-Square for the degradation of MB over the BiVO_4_ nanobelts were 0.02361 and 0.9679, respectively, indicating that the pseudo-first-order kinetic model can be used to describe the photodecomposition reactions of MB catalyzed by BiVO_4_ nanobelts.

Furthermore, the stability of the sample is one of the key factors to determine whether a photocatalyst has a promising future. In order to study the stability of monoclinic BiVO_4_ nanobelts, the solution of MB was degraded for three cycles with BiVO_4_ nanobelts as photocatalysts. The results of the reaction are shown in Figure 6c. With the increase in the number of cycles, the efficiency of photodegradation suffered a slight decrease, demonstrating that this BiVO_4_ nanobelt has high stability and be applied in practical MB degradation.

### 2.5. Possible Photocatalytic Mechanism

**Scheme 2:**
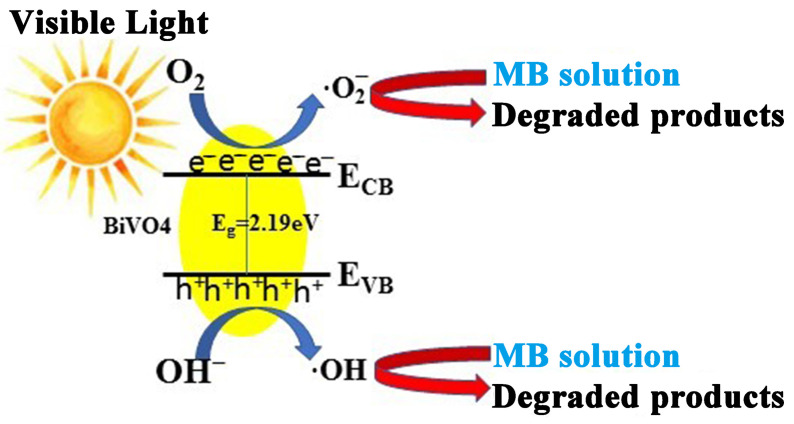
The photocatalysis mechanism for disintegrating MB catalyzed by BiVO_4_ nanobelts.

The superiority of the BiVO_4_ nanobelts is embodied in the ability to be used in photocatalysis. The UV–Visible diffuse reflectance spectrum of the BiVO_4_ nanobelts displayed significantly higher absorption performance in the visible range (Figure 3b), which may be attributed to crystal defects or quantum effects. The highest absorption wavelength of the BiVO_4_ nanobelts was approximately 565 nm while the band gap was calculated to be ~2.19 eV, which was narrower than that of the reported monocline BiVO_4_ crystal previously [15,24,30,31,32,33,34]. The Bi 6s and O 2p orbitals formed the valence band of a monocline BiVO_4_ crystal, which has been clearly certified by previous studies [30,31,35]. Moreover, its lower band gap and the mixed valence band of the obtained BiVO_4_ nanobelts can improve the transfer of photogenerated carriers. Hence, the BiVO_4_ nanobelts have excellent photocatalytic performance under the sun-like irradiation. Furthermore, the obtained BiVO_4_ nanobelts displayed outstanding photocatalytic properties in the area of organic pollutant degradation, in comparison to that of other single visible-light photocatalysts, suggesting that this BiVO_4_ nanobelt is thus a promising candidate for application in visible-light-responsive fields. To further investigate the photodegradation reactions of dye pollutants such as MB, the mechanism of photocatalytic activity over BiVO_4_ is described in Scheme 2.

Under the sun-like irradiation, the energy of the quantum photon was absorbed by the BiVO_4_ materials, the electrons transitioned from the BiVO_4_ VB to the CB, which produced photogenerated electrons, and photogenerated holes were formed on the VB due to electron deletion. Photogenerated electrons can reduce O_2_ to generate ·O_2_^−^, and photogenerated holes reduce OH^−^ to generate ·OH. Then, ·O_2_^−^ and ·OH with strong oxidation performance will lead to the photodegradation of MB. The photocatalytic degradation route of MB by BiVO_4_ nanobelts as photocatalysts may be as follows:

$$\begin{array}{l} {\mathrm{BiVO}}_{4}+hv\overset{}{\to }{\mathrm{BiVO}}_{4}(e^{-}+h^{+})\\ h^{+}+{\mathrm{OH}}^{-}\overset{}{\to }\cdot \mathrm{OH}\\ e^{-}+O_{2}\overset{}{\to }\cdot O_{2}^{-}\\ \mathrm{MB}\overset{h^{+},\cdot \mathrm{OH},\cdot O_{2}^{-}}{\to }{\mathrm{CO}}_{2}+H_{2}O+\mathrm{mineral}\;\mathrm{acids} \end{array}$$

Above all, the outstanding photocatalytic performance of the BiVO_4_ nanobelts is attributed to the synergy of the following two causes: (1) the small band gap of 2.19 eV can expand the absorption range of visible light and enhance the oxidation capability of the photoinduced carriers using the adsorbed OH¯ converted into ⋅OH radicals quickly; (2) the recombination chance of holes and electrons were largely decreased because of the rapid reaction process [23,36].

## 3. Materials and Methods

### 3.1. Chemicals

All chemicals were of analytical grade and purchased from Sinopharm Chemical Reagent Co., Ltd. (Shanghai, China); bismuth trioxide (Bi_2_O_3_, ≥99.0%), vanadic oxide (V_2_O_5_, ≥99.8%), polyethylene glycol 10000 (PEG 10000, %) and other chemicals were used without purification.

### 3.2. Preparation of One-Dimensional BiVO_4_ Nanobelts

Herein, 40 mL of hermetically sealed hydrothermal reactors with a polytetrafluoroethylene tank was used for the experiment. The self-generated pressure of hydrothermal system was always used to drive the diffusion and motion of material at medium temperature. And the synthetic procedure of BiVO_4_ nanobelts with PEG 10000 as template is described in detail as follows. Firstly, 1.86 g of Bi_2_O_3_ and 0.73 g of V_2_O_5_ were added in a clean and dry polytetrafluoroethylene container, respectively. Then, 0.4 g PEG 10000 was added with 32 mL redistilled water into the above mixture. Thirdly, the mixture was subjected to ultrasound for 20 min to obtain the homogeneous solution and then transferred into a Teflon-lined stainless-steel autoclave, reacted at 200 °C and kept for 24 h. The product was washed with deionized water, acetone and anhydrous ethanol 5–6 times, and then dried in vacuum at 60 °C for further characterization.

### 3.3. Product Characterization and the Visible-Light Catalysis Experiment

The FT-IR data of the obtained BiVO_4_ nanobelts were collected by a FT-IR spectrometer. The morphology was measured by a scanning electron microscope and a transmission electron microscope with HRTEM. The X-ray powder diffractometer (Bruker D8, Serqi Technology Co., Ltd, Bruker, German) was employed to determine the powder X-ray diffractograms (XRD) of the sample, A Cu Kα1 X-ray radiation source at 1.5406 Å was used for determination. The chemical composition and chemical state of the material surface were analyzed by X-ray photoelectron spectroscopy through Themo Scientific K-Alpha X-ray photoelectron spectrometer (USA) using an EX06 ion source. The photocatalytic activity of the prepared BiVO_4_ nanobelts was evaluated by degrading MB solution. The degradation rate of MB was determined by a UV–Vis spectrophotometer (U-3900H, Hitachi Co., Ltd., Tokyo, Japan).

### 3.4. Photoelectrochemical Measurement

The photoelectrochemical measurements were carried out on an electrochemical work station (CHI660E, Shanghai Chenhua Instrument Co., Ltd., Shanghai, China), using a standard three-electrode cell with the saturated Ag/AgCl electrode as a reference electrode. The platinum wire was used as the counter electrode while the as-prepared samples were used as the working electrode, which was obtained by pressing tablets. A 500 W xenon lamp was used as a light source to simulate visible light. The electrolyte used was 0.1 mol/L Na_2_SO_4_ aqueous solution.

The transitory photocurrent response of the as-prepared sample was recorded by an amperometry method under intermittent irradiation. Electrochemical impedance spectra (EIS) were obtained in the frequency range of 0.01–100,000 Hz.

## 4. Conclusions

In this paper, one-dimensional shaving-like BiVO_4_ nanobelts were successfully synthesized through the oxide hydrothermal method (OHS) using V_2_O_5_ and Bi_2_O_3_ as raw materials and PEG 10000 as a template. The BiVO_4_ nanobelts showed excellent optical performance and electrical function due to their unique appearance. The EIS results indicated that visible-light illumination made the photogenerated charge of BiVO_4_ nanobelts have a faster transfer and a higher separation efficiency, which was consistent with the photocatalytic activities. Moreover, photocatalytic experiments indicated that with BiVO_4_ nanobelts as a catalyst, the degradation rate of MB (methylene blue) was close to 92.4%, and it disintegrated after two hours, which was better than that of other single visible-light-driven catalysts. Moreover, the pseudo-first-order kinetic model can be used to describe the photodecomposition reactions of MB catalyzed by BiVO_4_ nanobelts. This study verifies that novel monoclinic BiVO_4_ nanobelts exhibited great photocatalytic activity and are thus promising candidates for application in visible-light-responsive fields.

## Data Availability

The data presented in this research are available on request from the corresponding author.
